# Constructing a gene semantic similarity network for the inference of disease genes

**DOI:** 10.1186/1752-0509-5-S2-S2

**Published:** 2011-12-14

**Authors:** Rui Jiang, Mingxin Gan, Peng He

**Affiliations:** 1 MOE Key Laboratory of Bioinformatics and Bioinformatics Division, TNLIST/Department of Automation, Tsinghua University, Beijing 100084, China; 2 School of Economics and Management, University of Science and Technology Beijing, Beijing 100083, China

## Abstract

**Motivation:**

The inference of genes that are truly associated with inherited human diseases from a set of candidates resulting from genetic linkage studies has been one of the most challenging tasks in human genetics. Although several computational approaches have been proposed to prioritize candidate genes relying on protein-protein interaction (PPI) networks, these methods can usually cover less than half of known human genes.

**Results:**

We propose to rely on the biological process domain of the gene ontology to construct a gene semantic similarity network and then use the network to infer disease genes. We show that the constructed network covers about 50% more genes than a typical PPI network. By analyzing the gene semantic similarity network with the PPI network, we show that gene pairs tend to have higher semantic similarity scores if the corresponding proteins are closer to each other in the PPI network. By analyzing the gene semantic similarity network with a phenotype similarity network, we show that semantic similarity scores of genes associated with similar diseases are significantly different from those of genes selected at random, and that genes with higher semantic similarity scores tend to be associated with diseases with higher phenotype similarity scores. We further use the gene semantic similarity network with a random walk with restart model to infer disease genes. Through a series of large-scale leave-one-out cross-validation experiments, we show that the gene semantic similarity network can achieve not only higher coverage but also higher accuracy than the PPI network in the inference of disease genes.

**Contact:**

ruijiang@tsinghua.edu.cn

## Background

Not withstanding the remarkable success of such statistical methods as linkage analysis and association studies in identifying genetic variants underlying inherited human diseases in the past few decades [1], susceptibility genomic regions obtained by these methods may contain dozens or even hundreds of candidate genes, appealing for the development of effective computational methods to infer genes that are truly associated with a query disease of interest from a long list of candidates [2].

In the face of this challenge, several methods have been proposed to score genes in a candidate list according to their functional relevance to the genes that are already known to be associated with the query disease (i.e., seed genes) and then prioritize the candidates according to their scores. The basic assumption of these methods, which is typically referred to as the “guilt-by-direct-association” principle, is that genes associated with a disease should have similar functions. It is therefore crucial for these methods to estimate functional similarity between genes. For this purpose, a wide variety of genomic information has been adopted, with examples including protein sequences [3], gene expression profiles [4], literature descriptions [5], protein-protein interactions (PPI) [6], gene ontology annotations [7], and many others [8]. Methods using multiple genomic data sources have also been proposed [9,10].

Depending on seed genes to prioritize candidate genes will restrict the scope of application of the above methods, because genetic bases for about half of the known human diseases are completely unknown according to the Online Mendelian Inheritance in Man (OMIM) database [11]. To overcome this limitation, recent studies have suggested the “guilt-by-indirect-association” principle, which relies on the modular nature of inherited human diseases [8,12] and resorts to a phenotype similarity network of diseases [13] to prioritize candidate genes [14-17,20]. These methods successfully extend the scope of prioritizing candidate genes to diseases whose genetic bases are completely unknown.

However, all methods based on the “guilt-by-indirect-association” principle thus far are designed to be used with one or more protein-protein interaction networks. For example, Wu et al. used a linear regression model to explain phenotype similarity using protein network proximity [15]. Zhang et al. extend the regression model to include multiple protein-protein interaction networks [19]. Li and Patra utilized a random walk model to simulate the steady-state probability of a random walker staying at a gene [17]. Although a protein-protein interaction network could provide a simplified yet systematic view of functional relationships between genes, the coverage of available protein-protein interaction networks is typically low, and the reliability of different protein-protein interaction networks is quite different [19], making the selection of a suitable network far from trivial. Moreover, focusing on common interactions in multiple networks to improve the confidence of edges will sacrifice the coverage of the resulting network, while focusing on the union of interactions to improve the coverage will result in a network of low reliability [19].

Motivated by these observations, we propose to construct a gene semantic similarity network using the biological process domain of gene ontology and GO annotations of human genes. We show that the gene semantic similarity network covers 14,085 genes, about 50% more genes than the widely used Human Protein Reference Database (HPRD) [21] protein-protein interaction network. Via a comprehensive analysis of the gene semantic similarity network with the HPRD network, we show that gene pairs tend to have higher semantic similarity scores if the corresponding proteins are closer to each other in the HPRD network. Through a detailed analysis of the gene semantic similarity network with a phenotype similarity network, we show that semantic similarity scores of genes associated with similar diseases are significantly different from those of genes selected at random, and that genes with higher semantic similarity scores tend to be associated with diseases with higher phenotype similarity scores. We further use the gene semantic similarity network with a random walk with restart model [17] to infer disease genes. Through a series of large-scale leave-one-out cross-validation experiments, we show that the gene semantic similarity network can achieve not only higher coverage but also higher accuracy than the HPRD network in the inference of disease genes. With these results, we conjecture that the gene semantic similarity network can serve as a better assessment of functional relationship between genes and then be used in a large number of applications in systems biology.

## Results

### Data sources

We propose to prioritize candidate genes using 1) a gene semantic similarity network that is constructed using the biological process (BP) domain of the gene ontology (GO) and known GO annotations of human proteins, 2) a phenotype similarity network of human diseases, and 3) known associations between diseases and genes.

First, we extract 18, 850 GO terms in the biological process domain from the gene ontology (released on April 18, 2010) and extract 186, 080 annotations of human proteins from the UniProtKB GO annotations of human proteins (released on April 18, 2010). Focusing on proteins with corresponding gene identifiers in the Ensembl database, we obtain 59,681 annotations that involve 14,085 human genes and 5,596 GO terms.

Second, we obtain a phenotype similarity profile, represented as a matrix of similarity scores between 5,080 human diseases, from the literature [13]. Since most small similarity scores in this profile are likely to be noise and only high scores have clear biological meanings [13], we follow the literature [17] to keep the first five nearest neighbors for each disease and obtain a phenotype similarity network, in which vertices are human diseases and weighted edges indicate similarity scores between diseases.

Third, we use the tool BioMart [22] to extract 4, 368 known associations that involve 2,593 human genes with Ensembl gene identifier and 3, 111 human diseases in the OMIM database [11].

Finally, we use the high quality Human Protein Reference Database (HPRD) [21] to demonstrate the relationship between a gene semantic similarity network and a protein-protein interaction network. After removing duplications and self-linked interactions, we extract from release 9 (release on April 13, 2010) of this database 37, 067 interactions between 9,518 human genes.

### Construction of gene semantic similarity networks

**Figure 1:**
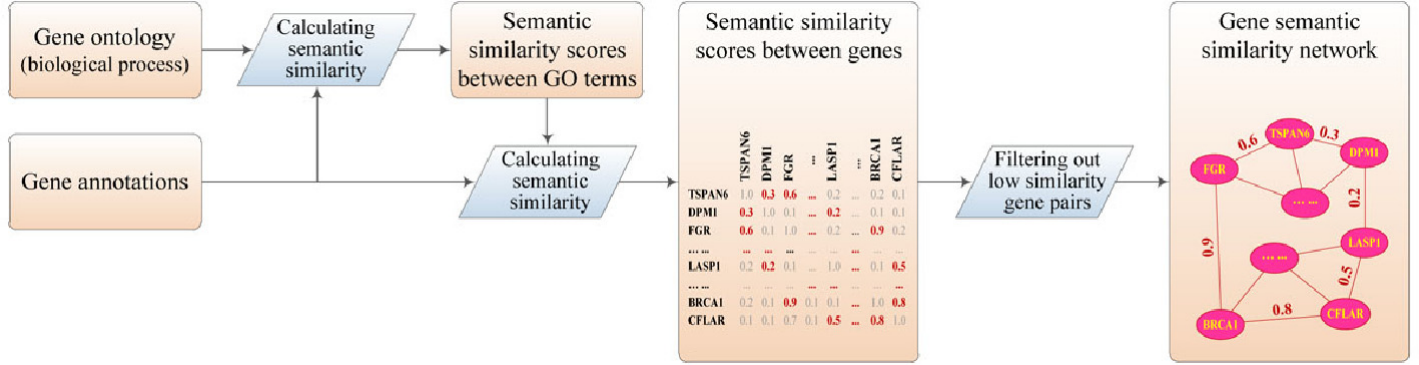
Illustration of the procedure for constructing a gene semantic similarity network.

The procedure of constructing a gene semantic similarity network is illustrated in Figure 1. First, we calculate pairwise semantic similarity scores for GO terms in the biological process domain, obtaining a matrix that contains semantic similarity scores between GO terms. Next, we calculate pairwise semantic similarity scores for human genes using similarity scores of GO terms and annotations of genes, obtaining a matrix that contains semantic similarity scores between genes. Then, we filter out low similarity values in this matrix by keeping only the first *κ* nearest neighbors for each gene and assigning zeros to all other elements. Finally, we obtain a gene semantic similarity network by treating non-zero elements in the resulting matrix as weights of edges between corresponding genes.

We adopt three methods based on information contents of GO terms (Resnik [23], Schlicker et al. [24] and Lin [25]) and one method based on the structure of gene ontology (Wang et al. [26]) to calculate similarity scores for GO terms, and we use a method in the literature [26] to calculate similarity scores for genes (see Methods for details). Hence, we obtain four semantic similarity networks, each containing 14,085 human genes.

### Gene semantic similarity correlates with protein network proximity

There have been a few methods relying on protein-protein interaction networks to infer disease genes [17]. The basic assumption of these methods is that interacting proteins are usually related in their functions, and thus the proximity of two proteins in a protein-protein interaction network can be used as an estimation of the functional relationship between the corresponding genes. Therefore, we first show that the similarity score between two genes in a gene semantic similarity network correlates with the proximity score of the corresponding proteins in a protein-protein interaction network.

**Figure 2:**
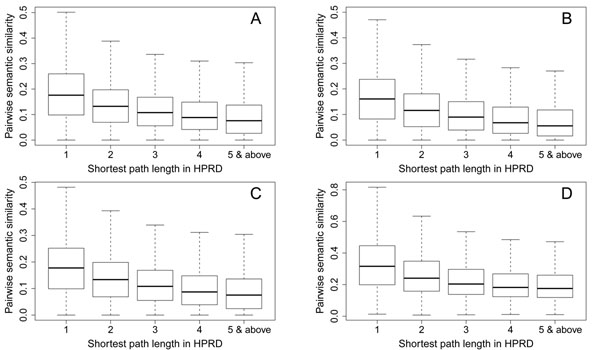
Relationship of gene similarity scores and protein network proximity scores. A: results for the method of Resnik. B: results for the method of Schlicker et al. C: results for the method of Lin. D: results for the method of Wang et al.

We use the length of the shortest path between two proteins in the HPRD network to measure their proximity, and we draw box plots to demonstrate the relationship between gene semantic similarity scores and protein network proximity scores in Figure 2. From the figure, we can clearly see that gene pairs tend to have higher semantic similarity scores if the corresponding proteins are closer in the protein-protein interaction network. Taking gene semantic similarity scores calculated using the method of Resnik as an example (Figure 2:A), the median semantic similarity score is 0.1760 for gene pairs whose products have direct interaction in HPRD, 0.1322 for gene pairs intermediated by another gene in HPRD, 0.1028 for gene pairs intermediated by two other genes, 0.0830 for gene pairs intermediated by three other genes, and 0.0698 for gene pairs intermediated by four or more other genes. Similar results are observed for gene semantic similarity scores calculated using the other methods.

These results suggest that gene semantic similarity scores are correlated with protein proximity scores. Hence, given the successful applications of [14-20], it is reasonable to use gene semantic similarity networks for the inference of disease genes.

### Gene semantic similarity implies disease phenotype similarity

**Figure 3:**
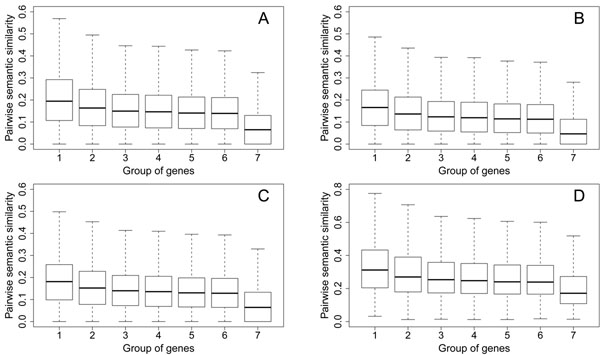
Pairwise semantic similarity scores of genes in different groups. A: results for the method of Resnik. B: results for the method of Schlicker et al. C: results for the method of Lin. D: results for the method of Wang et al.

The phenotype similarity profile of diseases has been successfully used for prioritizing candidate genes in recent studies [14-20]. In general, methods relying on the phenotype similarity profile assume that similar diseases are associated by genes with similar functions. It is therefore necessary to assess whether semantic similarity scores between genes associated with similar diseases are significantly different from those between genes that are selected at random. For this purpose, we partition genes into 7 groups according to the similarity scores of diseases that the genes are associated, and we draw the box plot of pairwise similarity scores of genes in each group in Figure 3.

We use gene semantic similarity scores calculated using the method of Resnik as an example to demonstrate the relationship between gene semantic similarity and disease phenotype similarity (Figure 3:A). In group 1, we look at each disease separately. We collect genes that are associated with a disease, plot pairwise semantic similarity scores of these genes, and obtain a median semantic similarity score of 0.1945 for this group of genes. In group 2, we look at the nearest neighbor (the disease with the highest similarity score) of each disease in the disease similarity network. We collect genes associated with a disease and genes associated with the nearest neighbor of the disease, and we obtain a median pairwise semantic similarity score of 0.1635 for this group of genes. In group 3, we look at the second nearest neighbor of each disease in the disease similarity network. We collect genes that are associated with a disease and its second nearest neighbor, and we obtain a median pairwise semantic similarity score of 0.1486 for this group of genes. Similarly, in groups 4, 5 and 6, we look at the third, fourth and fifth nearest neighbor of each disease, respectively, and we obtain median pairwise semantic similarity scores of 0.1441, 0.1394 and 0.1383 for the corresponding groups of genes, respectively. Finally, in group 7, we look at 10,000 pairs of genes that are selected at random, and we obtain a median pairwise semantic similarity score of 0.0649.

These results demonstrate that semantic similarity scores of genes associated with similar diseases are significantly different from those of genes selected at random, and that genes with higher semantic similarity scores tend to be associated with diseases with higher phenotype similarity scores. In other words, semantic similarity of genes implies phenotype similarity of diseases that the genes are associated.

### Gene semantic similarity networks improve the accuracy in prioritizing candidate genes

**Figure 4:**
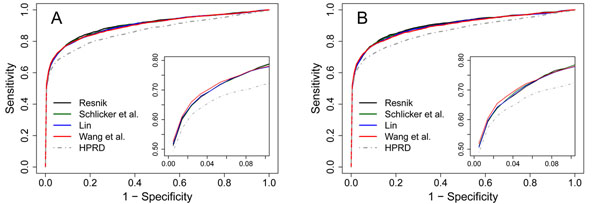
ROC curves of the proposed approach. A: results for the validation of a linkage-interval. B: results for the validation of random genes.

We propose to prioritize candidate genes using a gene semantic similarity network, the phenotype similarity network, and known associations between diseases and genes. This is done by applying a random walk with restart model to a heterogeneous network that is composed of both diseases and genes (see Methods). We adopt two large-scale leave-one-out cross-validation experiments with two comprehensive evaluation criteria to assess the performance of this approach (see Methods), and we present results in Table 1 and Figure 4.

**Table 1:** Performance of the semantic similarity networks and the HPRD network in the validation experiments. Candidate genes are selected from the overlap of the semantic similarity and the HPRD networks.

	Resnik (%)	Schlicker (%)	Lin (%)	Wang (%)	HPRD (%)
Linkage interval					
MRR	10.60	10.86	10.97	11.05	14.21
AUC	90.30	90.04	89.93	89.85	86.65
Random genes					
MRR	10.65	10.92	11.06	11.20	14.40
AUC	90.25	89.98	89.84	89.70	86.46

We use the gene semantic similarity network constructed using the method of Resnik as an example to demonstrate the performance of the proposed approach. At the threshold *κ* = 100, we obtain a network composed of 14, 085 genes and 2,112, 750 edges. Taking the overlap of genes in this network and those in the HPRD database, we obtain 8, 286 genes. Focusing on these genes, we obtain 2,397 associations between 1,572 diseases and 1,391 genes. We then perform the leave-one-out cross-validation experiment against a linkage interval and obtain the Mean Rank Ratio of disease genes (MRR) as 10.60% and the Area Under the rank receiver characteristic Curve (AUC) as 90.30%. We further perform the validation experiment against random genes and obtain an MRR of 10.65% and an AUC of 90.25%. Since a random guess will yield an MRR of 50% and an AUC of 50%, these results clearly suggest the effectiveness of relying on the semantic similarity network to uncover disease genes. For gene semantic similarity networks constructed using the other methods, we obtain similar results (Table 1).

We replace the gene semantic similarity network with the HPRD network and repeat the experiments. In the validation of a linkage interval, we obtain an MRR of 14.21% and an AUC of 86.65%. In the validation of random genes, we obtain an MRR of 14.40% and an AUC of 86.46%. We further plot the ROC curves of the validation results in Figure 4, from which we observe that the curves for the gene semantic similarity networks climb much faster towards the top left corner of the plot than that for the HPRD network. From these results, we conclude that the gene semantic similarity networks are superior to the HPRD network in the prioritization of candidate genes.

**Figure 5:**
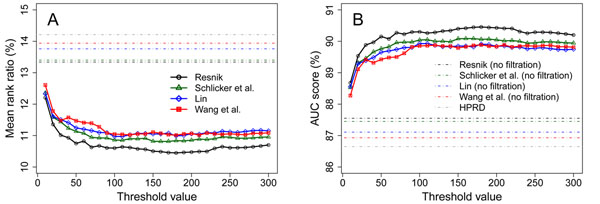
Influence of the parameter *κ* to the performance of the random walk model in the validation of a linkage interval. Solid lines represent criteria obtained at different *κ* values. Dot-dash lines represent baseline values of the criteria.

We assess the influence of the threshold *κ* on the performance of the random walk model. We vary this parameter from 10 to 300 with step 10, perform the validation against a linkage interval at each value, and present the results in Figure 5. First, we observe that a relatively small *κ* for filtering out low semantic similarity scores will improve the performance of the prioritization method. For example, with the use of the semantic similarity network constructed using the method of Resnik, we obtain an MRR of 13.36% and an AUC of 87.51% when using all similarity scores without filtration (corresponding to *κ* ≥ 14, 085). However, when using *κ* = 100, we obtain an MRR of 10.60% and an AUC of 90.30%, indicating a significant improvement against the results without filtration. Second, we observe that the prioritization method is not sensitive to this parameter when it is relatively small (compared with the number of genes in the network). For example, when using the method of Resnik, both the MRR and the AUC are stable when 100 ≤ *κ* ≤ 300. The optimal value of *κ* in this interval is 180, at which we obtain an MRR of 10.45% and an AUC of 90.46%, only slightly better than the results at *κ* = 100. This property is important to the selection of the parameter *κ*. More specifically, since the performance of the prioritization method is only slightly affected by *κ* when it is relatively small, we can roughly select a *κ* value to obtain near optimal performance. Hence, we default *κ* to 100 in the rest of this paper unless declaring explicitly.

### Gene semantic similarity networks improve the coverage in prioritizing candidate genes

The reliability and coverage of existing protein-protein interaction data sets are quite different. Focusing on common interactions in these data sets to improve the confidence will sacrifice the coverage; considering the union of interactions to improve the coverage will result in a network of low reliability. A gene semantic similarity network, however, can cover a large proportion of human genes while providing high accurate inference of disease genes.

We focus on the network constructed using the method of Resnik to demonstrate the effectiveness of relying on gene semantic similarity networks to infer disease genes. At the threshold *κ* = 100, we obtain a network composed of 14,085 genes and 2,112,750 edges. Focusing on these genes, we obtain 3,047 associations between 1,984 diseases and 1,877 genes. We then perform cross-validation experiments against random genes and a linkage interval and present the results in Table 2. In the validation against a linkage interval, we obtain an MRR of 10.41% and an AUC of 90.50%. In the validation against random genes, we obtain an MRR of 10.19% and an AUC of 90.72%. These results clearly suggest the high accuracy of relying on the gene semantic similarity network to infer disease genes.

**Table 2:** Performance of the semantic similarity networks in the validation experiments. Candidate genes are selected from the semantic similarity networks.

	Resnik (%)	Schlicker (%)	Lin (%)	Wang (%)
Linkage interval				
MRR	10.41	10.70	10.84	10.95
AUC	90.50	90.20	90.06	89.95
Random genes				
MRR	10.19	10.48	10.62	10.79
AUC	90.72	90.42	90.68	90.11
Random genes (999)				
MRR	10.14	10.49	10.60	10.81
AUC	90.36	90.01	89.91	89.69
Genome-wide scan				
MRR	10.16	10.49	10.60	10.81
AUC	90.10	89.77	89.66	89.45

We further increase the number of random genes in each validation run to 999 and find the AUC only drop slightly to 90.36%, suggesting that the prioritization method is not sensitive to the number of control genes in validation. With this understanding, we pursue a more ambitious goal of genome-wide scan for disease genes and obtain an MRR of 10.16% and an AUC of 90.10% in uncovering the disease genes from all 14,085 genes in the gene semantic similarity network.

**Figure 6:**
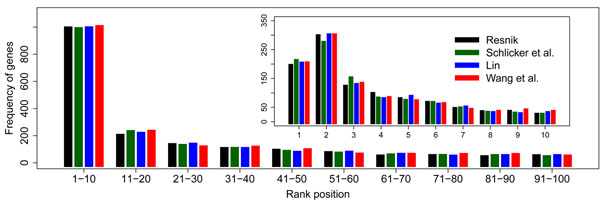
The distribution of genes ranked in top 100 in the genome-wide scan of disease genes.

We then look at in detail the distribution of disease genes ranked within top 100 of the 14, 085 genes and present the results in Figure 6. We observe that 1, 602 (52.58%) diseases genes are ranked in top 100 when relying on the network constructed using the method of Resnik. Within these disease genes, 974 (31.97%) are ranked in top 10, 182 (5.97%) ranked between 11 and 20, 114 (3.74%) ranked between 21 and 30, 85 (2.79%) ranked between 31 and 40, and 72 (2.36%) ranked between 41 and 50. In the zoomed-in plot of Figure 6, we observe 192 (6.30%) disease genes ranked first, 295 (9.68%) ranked second, 120 (3.94%) genes ranked third, 95 (3.12%) genes ranked fourth, and 77 (2.53%) genes ranked fifth. Furthermore, we find that the logarithm of the number of genes at a rank fits a linear model with the rank (log(#{genes}) = 5.72 *–* 0.26 × rank), and the model is statistically significant with a *r*^2^ of 0.9383 and a *p*-value of 4.059 × 10^–6^. These results suggest the effectiveness of relying on the gene semantic similarity network to scan genes potentially associated with a query disease from the whole genome. Particularly, for query diseases whose genetic bases completely unknown (and thus no linkage information is available), researchers can relying on the semantic similarity network to perform a genome-wide scan and then focus on top ranked genes to narrow down the scope of searching for disease genes.

We also notice that relying on semantic similarity networks constructed using the other methods (with default threshold values) yields similar results as we analyzed above (Table 2).

## Conclusions and discussion

In this paper, we have proposed to rely on the biological process domain of gene ontology and GO annotations of human genes to construct a semantic similarity network of genes, and then use the network with phenotype similarity network of diseases to infer genes that are associated with a query disease of interest.

The main objective of this research is to overcome one of the shortcomings of existing protein-protein interaction networks, i.e., the low coverage. The constructed gene semantic similarity network covers 14,085 genes, about 50% more than the widely used HPRD network. More importantly, as demonstrated in our comprehensive analysis, the improvement in coverage is accompanied by the gain in accuracy in the inference of disease genes. Hence, the gene semantic similarity network can serve as a better assessment of functional relationship between genes and then be used in a large number of applications in systems biology.

The filtration of low semantic similarity scores is important to the success of the proposed approach. We currently achieve this goal by keeping the first *κ* nearest neighbors of each gene. Alternatively, we can introduce a threshold and discard all edges whose weight (similarity score) is less than the threshold. According to our experiments, this alternative strategy is likely to yield a disconnected network and thus adversely affect the performance of a prioritization method relying on the network. Therefore, we resort to the nearest neighbor strategy to filter out low semantic similarity scores.

Certainly, our research can further be improved from the following aspects. First, although we have focused on the biological process domain in this paper, it is conceptually straightforward to use the molecular function and the cellular component domains to construct gene semantic similarity networks. According to our experiments, semantic similarity networks relying on these two gene ontology domains have similar coverage as that of the biological process domain and can achieve comparable performance as the HPRD network in the inference of disease genes (data not shown). Therefore, a possible improvement of our approach is to construct a gene semantic similarity network with the integration of all three domains in the gene ontology.

Second, the semantic similarity network and the protein-protein interaction network assess the functional relationship between genes from different points of view. Therefore, the inference of disease genes may be benefit from the integrated use of these two types of networks. Furthermore, as the effectiveness of relying on the “guilt-by-association” principle (without using the phenotype similarity profile) and multiple genomic data to infer disease genes has been demonstrated in previous studies. It is reasonable to pursue the goal of using the phenotype similarity profile with multiple genomic data to achieve more accurate inferences of disease genes.

## Methods

### Calculation of semantic similarity scores

We adopt three methods based on information contents of GO terms (Resnik [23], Schlicker et al. [24] and Lin [25]) and one method based on the structure of gene ontology (Wang et al. [26]) to calculate semantic similarity scores between GO terms.

Given the gene ontology and annotations of human genes, the probability of occurrence of a GO term *t* in annotations, *p*(*t*), is estimated as the number that the term or its descendants are used in annotations divided by the total number of annotations, as

In general, more specific terms are less frequently used in annotations and thus have lower probability of occurrence. A pair of terms *a* and *b* usually has more than one common ancestor in the ontology. Let  be the set of all common ancestors of *a* and *b*, the probability of occurrence of the most concrete common ancestor of *a* and *b* is then calculated as

With these definitions, the method of Resnik [23] calculates the semantic similarity score between two terms *a* and *b* as the information content (negative logarithm of the probability) of the most concrete common ancestor of the two terms, as

The method of Lin [25] normalizes the above information content with the average information content of the two terms, as

The method of Schlicker et al. [24] further weights the above quantity with the probability of occurrence of the most concrete common ancestor of the two terms, as

Different from the above methods that rely on annotations of genes, the method of Wang et al. [26] depends only on the structure of gene ontology to calculate semantic similarity scores between GO terms. Let *t* be a GO term,  the set of its ancestors and  the set of its children in the GO structure. Wang et al. iteratively calculate an *s*-value for every term  to measure the contribution of *a* to the semantics of *t*, as

where the weight factor *w_e_* = 0.8 if *x* and *a* have the “is_a” relationship and *w_e_* = 0.6 if *x* and *a* have the “part_of” relationship. Then, a semantic value for a term *t* is calculated as

Finally, the semantic similarity score between two terms *a* and *b* is calculated as

With the semantic similarity scores between GO terms calculated by either of the above methods, we calculate the semantic similarity between two genes as follows. The semantic similarity score between a GO term *t* and a set of GO terms  is calculated as

The semantic similarity score between two sets of GO terms  and  is calculated as

Let *g* and *g′* be two genes. Let  and  be the two sets of GO terms with which *g* and *g′* are annotated, respectively. The semantic similarity between *g* and *g′* is then calculated as

Applying the above method to every pair of genes, we obtain a pairwise semantic similarity matrix of genes. Certainly, this matrix can be thought of as the weight matrix of a fully connected network, whose vertices are genes and whose edges represent semantic similarity scores between genes. However, such a fully connected network may contain a large number of low confident edges between gene pairs with low semantic similarity scores. We therefore further filter out edges with low weights (similarity scores) in the fully connected network by introducing a threshold *κ* (defaulting to 100 in this paper) and keeping only the first *κ* nearest neighbors for each gene. By doing this, we obtain a gene semantic similarity network.

### Prioritization of candidate genes

The random walk with restart on the heterogeneous network model [17] is one of the state-of-the-art methods that utilize a disease similarity network with a protein-protein interaction network to prioritize candidate genes. This model simulates the process that a random walker wanders on a heterogeneous network composed of a phenotype similarity network, a protein-protein interaction network, and known associations between diseases and genes. In each step of the process, the random walker may start on a new journey with probability *γ* or move on with probability 1 – *γ*. When starting on, the walker may choose the query disease of interest as the starting point with probability *η* or choose a seed gene known to be associated with the query disease with probability 1 – *η.* When moving on, the walker may choose to jump from the disease similarity network to the protein-protein interaction network or vice versa with probability *λ* or choose to wander in either the disease network or the protein-protein interaction network with probability 1 – *λ*. When wandering about, the walker moves at random to one of its direct neighbors.

In this model, the protein-protein interaction network serves as a simplified yet systematic view of functional relationships among genes. Since a gene semantic similarity network also provides a means of measuring functional relationships among genes, conceptually we can also use a gene semantic similarity network with the phenotype similarity network to infer disease genes. Following the literature [17], we use the following random walk with restart model on the heterogeneous network that is composed of a phenotype similarity network, a gene semantic similarity network, and known associations between diseases and genes.

We represent the phenotype similarity network using a weight matrix **D** = (*d_ij_*)*_m_*_×_*_m_*, where *m* denotes the number of diseases and *d_ij_* the similarity score between the *i*-th disease and the *j*-th disease. By normalizing each row of this matrix, we obtain a transition matrix **U** = (*u_ij_*)*_m_*_×_*_m_*, where , representing the probability that a random walker moves from the *i*-th disease to the *j*-th disease.

We represent the gene semantic similarity network using a weight matrix **G** = (*g_ij_*)*_n_*_×_*_n_*, where *n* denotes the number of genes and *g_ij_* the similarity score between the *i*-th gene and the *j*-th gene. By normalizing each row of this matrix, we obtain a transition matrix **V** = (*v_ij_*)_*n*×*n*_, where , representing the probability that a random walker moves from the *i*-th gene to the *j*-th gene.

We represent known associations between diseases and genes using an adjacency matrix **A** = (*a_ij_*)_*m*×*n*_, where *a_ij_* = 1 indicates that the *j*-th gene is known to be associated with the *i*-th disease, and *a_ij_* = 0 otherwise. By normalizing each row of this matrix, we obtain a transition matrix **R** = (*r_ij_*)_*m*×*n*_, where , representing the probability that a random walker jumps from the *i*-th disease to the *j*-th gene. Note that we define *r_ij_* = 0 when , i.e., when there is no gene known as associated with the *i*-th disease. Similarly, by normalizing each row of the transpose of the matrix **A**, we obtain a transition matrix **S** = (*s_ij_*)_*n*×*m*_, where , representing the probability that a random walker jumps from the *i*-th gene to the *j*-th disease. We also define *s_ij_* = 0 when  i.e., when the *i*-th gene is not associated with any disease.

With the above four transition matrices, we define

and further normalize every row of this matrix to obtain the transition matrix of the heterogeneous network **W** = (*w_ij_*), where . The parameter *λ* is the probability that the random walker jumps from the disease similarity network to the gene semantic similarity network or vice versa.

When the random walker starts in the disease similarity network, we let it start from the query disease, therefore the initial probability is 1 for the query disease and 0 for other diseases. We use a vector **u**^(0)^ to represent these probabilities. When the random walker starts in the gene similarity network, we let it start at random from one of the genes known as associated with the query disease, therefore the initial probability is 1*/s* for every seed gene (suppose there are a total of *s* seed genes) and 0 for other genes. We use a vector **v**^(0)^ to represent these probabilities. Let *η* be the probability that the random walker starts from the disease similarity network, we have the initial probability vector

Finally, let **p**^(^*^t^*^)^ be the vector composed of probabilities of finding the random walker at all vertices in the heterogeneous network at step *t*, we have

After a number of steps, the probability will reach a steady state. This is obtained by performing the iteration until the difference between **p**^(*t*)^ and **p**^(*t*+1)^ is sufficiently small (i.e., the *L*_1_ norm of Δ**p** = **p**^(*t*+1)^ – **p**^(*t*)^ is less than a small positive number *ε*). The steady-state probability **p**^(∞)^ then gives a measure of the strength of association of each gene to the query disease of interest, and we can then rank candidate genes according to their steady-state probabilities.

It has been show that the random walk model is not sensitive to the parameters involved in the model [17]. Hence, we follow the literature [17] and default the parameters to *λ* = 0.7, *η* = 0.5, *γ* = 0.5 and *ε* = 10^–4^.

### Validation methods and evaluation criteria

We perform three large-scale leave-one-out cross-validation experiments to examine the performance of the proposed method in prioritizing genes that are known to be associated with certain diseases (i.e., disease genes) from a set of candidates. First, in the validation against a linkage interval, we take a known association between a gene and a disease in each run, assume the association is unknown, and prioritize the gene against a set of 99 control genes that locate nearest to the disease gene according to their genomic distance on the same chromosome. Second, in the validation against random genes, we select control genes in each validation run as 99 (or 999) genes that are selected at random from all genes in a gene semantic similarity network. Third, in the genome-wide scan of disease genes, we select control genes in each validation run as all genes in a gene semantic similarity network.

We use two measures to evaluate the performance of the proposed method. Taking the cross-validation against a linkage interval as an example, after each validation run, we obtain a score (the steady-state probability) for each candidate gene and further rank genes according to their scores (ties are broke by assigning ranks to genes with equal scores at random) to obtain a ranking list of candidate genes. We then calculate rank ratios of candidate genes by dividing their ranks with the number of candidate genes in the list. For a set of validation runs, we calculate the following two measures. First, we calculate the mean rank ratio (MRR) of all disease genes as the average of rank ratios of all disease genes in the validation runs. Second, given a threshold of rank ratio, we calculate the sensitivity as the fraction of disease genes ranked above the threshold and the specificity as the fraction of control genes ranked below the threshold. Varying the threshold value from 0.0 to 1.0, we are able to draw a receiver operating characteristic (ROC) curve and further calculate the area under this curve (AUC). Obviously, smaller MRR and larger AUC values indicate higher performance of a prioritization method.

## Authors' contributions

RJ designed the research, collected the results and wrote the paper. MG analyzed the results and wrote the paper. PH analyzed the results. All authors read and approved the final manuscript.

## Competing interests

The authors declare that they have no competing interests.
